# Severe depression and all-cause and cause-specific mortality in Scotland: 20 year national cohort study

**DOI:** 10.1192/bjo.2023.633

**Published:** 2024-01-11

**Authors:** Raied Alotaibi, Nynke Halbesma, Sarah H. Wild, Caroline A. Jackson

**Affiliations:** Usher Institute, University of Edinburgh, Edinburgh, UK; and Prince Sultan College for Emergency Medical Services, King Saud University, Riyadh, Saudi Arabia; Usher Institute, University of Edinburgh, Edinburgh, UK

**Keywords:** Depressive disorders, mortality, epidemiology, mental health services, clinical outcomes measures

## Abstract

**Background:**

Understanding cause of death in people with depression could inform approaches to reducing premature mortality.

**Aim:**

To describe all-cause and cause-specific mortality for people with severe depression in Scotland, by sex, relative to the general population.

**Method:**

We performed a retrospective cohort study, using psychiatric hospital admission data linked to death data, to identify adults (≥18 years old) with severe depression and ascertain cause-specific deaths, during 2000–2019. We estimated relative all-cause and cause-specific mortality for people with severe depression using standardised mortality ratios (SMRs), stratified by sex using the whole Scottish population as the standard.

**Results:**

Of 28 808 people with severe depression, 7903 (27.4%) died during a median follow-up of 8.7 years. All-cause relative mortality was over three times higher than expected (SMR, both sexes combined: 3.26, 95% CI 3.19–3.34). Circulatory disease was the leading cause of death, and, among natural causes of death, excess relative mortality was highest for circulatory diseases (SMR 2.51, 2.40–2.66), respiratory diseases (SMR 3.79, 3.56–4.01) and ‘other’ causes (SMR 4.10, 3.89–4.30). Among circulatory disease subtypes, excess death was highest for cerebrovascular disease. Both males and females with severe depression had higher all-cause and cause-specific mortality than the general population. Suicide had the highest SMR among both males (SMR 12.44, 95% CI 11.33–13.54) and females (22.86, 95% CI 20.35–25.36).

**Conclusion:**

People with severe depression have markedly higher all-cause mortality than the general population in Scotland, with relative mortality varying by cause of death. Effective interventions are needed to reduce premature mortality for people with severe depression.

Monitoring the burden and mortality of physical and mental health disorders is essential for evaluating and improving the quality of healthcare and guiding policies at national and international levels.^1^ The Global Burden of Disease Study 2019 estimated that around 280 million people worldwide were affected by depressive disorders.^2^ Depression is associated with increased occurrence of and poor outcomes from physical disease and is linked to premature mortality,^3,4^ a disparity that may have widened over time.^5^ Cardiovascular disease (CVD) in particular has a bidirectional association with depression and is a major contributor to premature mortality in people with depression.^3,4^

Although many previous studies have examined the association between mental illness and premature death, identifying an excess risk of death from natural causes including CVD, respiratory disease and cancer,^6–8^ most did not stratify by specific type of mental disorders. In Scotland, there has been limited research on the association between depression and all-cause and cause-specific mortality. One previous study reported on the mortality experience of people with a hospital admission record for severe mental illness (SMI) from 1986 to 2009 and did differentiate between disorders.^9^ The authors reported that age-standardised all-cause mortality was 78% higher in people with depression compared with the general Scottish population. They identified CVD as the most common natural cause of death in people with depression but reported on cause-specific mortality rates for three death categories only (CVD, cancer and suicide) and did not stratify results by sex. Recent studies suggest possible sex differences in the contribution of cause-specific deaths to the shorter life expectancy of people with mental illness, including depression.^7,8,10^ Moreover, investigations of how associations might vary across subgroups of circulatory disease have been limited,^7^ with no investigation of this for depression specifically. Such insight would add valuable depth to our understanding of the excess burden of cardiovascular death among people with depression.

In the current study, we sought to add to the existing literature in this area by examining in detail the sex-specific associations between severe depression and all-cause and cause-specific mortality, with greater focus on subtypes of CVD causes of death then in previous studies and using more contemporary data from Scotland. Our primary aim was to describe the all-cause and cause-specific mortality experience of people with severe depression in Scotland separately for males and females, relative to the general Scottish population.

## Method

We conducted a retrospective cohort study using Scottish population-level data. We followed the RECORD (reporting of studies conducted using observational routinely collected health data) guidelines for reporting observational studies.^11^

### Identification and definition of severe depression

We defined severe depression based on diagnoses recorded in psychiatric hospital admission records. We included all people aged 18 years or older who had at least one psychiatric hospital admission record for depression in Scotland between 1 January 2000 and 31 December 2019. Participants were identified from the Scottish Mental Health Inpatient and Day Case (SMR04) data-set^12^ using diagnosis codes based on ICD-10 codes F32–F33.^13^ We obtained demographic and other information for participants including age at admission, sex, and admission date from the hospital records.

### Comparison group

We used the general Scottish population as our reference population. Scottish population data used in our analysis are publicly available on the National Records of Scotland (NRS) website. We used mid-year Scottish population estimates for the corresponding years, as well as vital events tables that include total numbers of deaths by sex, age and cause of death based on ICD-10 codes (https://www.nrscotland.gov.uk/statistics-and-data).

### All-cause and cause-specific mortality

Hospital admission data and mortality data in Scotland were linked by the electronic Data Research and Information Service of NHS Scotland using a unique community health index number assigned at birth or at registration with a general practitioner in Scotland. The primary outcome measures were all-cause mortality and cause-specific mortality. We categorised causes of death based on the most frequently recorded natural and unnatural causes of death in our study population's death certificates as follows: circulatory diseases (ICD-10 I00–I99); neoplasms (ICD-10 C00–D48); respiratory diseases (ICD-10 J00–J99); mental and behavioural disorders (ICD-10 F00–F99); accidents (unintentional injuries; ICD-10 V01–X59, Y85–Y86); suicide, self-harm and injuries of undetermined intent (ICD-10 X60–X84, Y10–Y34, Y870, Y872); and all other natural causes of death.^14^ The secondary outcome of this study was mortality from subcategories of circulatory diseases, defined as: ischaemic heart disease (IHD) (ICD-10 I20–I25); cerebrovascular disease (ICD-10 I60–I69); other cerebrovascular diseases (ICD-10 I100–I13, I26–I51); and other CVD (ICD-10 I70–I99; Supplementary Table 1 available at https://doi.org/10.1192/bjo.2023.633).

### Statistical analysis

We calculated person-time at risk for people with severe depression by sex, 5 year age group and calendar-year group, taking into account each individual's time-at-risk across different age and calendar-year groups. Cohort entry date was specified as the date of the first recorded depression admission to a psychiatric hospital, with the end date being the earliest of date of death or end of the study period (31 December 2019) for each person.

We quantified the relative difference in mortality by calculating the standardised mortality ratio (SMR) for males and females, using the indirect standardisation method.^15^ The SMR in the indirect method was calculated by dividing the observed number of deaths in individuals with severe depression by the expected number of deaths, which was determined by applying mortality rates from the general Scottish population to the corresponding calendar year, age and sex-specific time at risk of the cohort with severe depression.^15^

We used NRS tables to calculate the standard mortality rates for the Scottish population for each age group, sex, calendar year and cause of death. SMR was standardised for sex and age in 5 year bands (18–22, 23–27, 28–32, etc. up to 82+ years) and by 5 year calendar periods (2000–2004, 2005–2009, 2010–2014 and 2015–2019).

We performed a sensitivity analysis to explore the impact of excluding individuals with other admission records for schizophrenia (ICD-10, F20–F29), bipolar disorder (ICD-10, F30–F31) and drug or alcohol-induced psychotic disorder (ICD-10, F10–F19) from the cohort of people with severe depression to provide data comparable with that of the previous Scottish study.^9^

We used Stata 16.0 MP for all analyses, with the istdize set of commands used to estimate SMRs with 95% confidence intervals.^16^

### Ethics statement

Permission to conduct this study with pseudonymised, non-consented data was obtained from the Public Benefit and Privacy Panel of National Health Service National Services Scotland (reference number 1516-0626).

## Results

### All-cause and cause-specific mortality

We included 28 837 people who had a diagnosis of depression recorded in psychiatric hospital admission data in Scotland between 1 January 2000 and 31 December 2019. The median (interquartile range [IQR]) age at admission was 45 (32–64) years, with just over half of participants being female (55.3%). The study population was followed up for a total of 264 172 person-years, with a median (IQR) follow-up time of 8.7 (3.8–14.3) years. In total, 7903 (27.4%) people died during follow-up, at a median (IQR) age of 76 (59–84) years. Age at death was slightly younger in males than in females (Table 1).

**Table 1:** Characteristics of people aged ≥18 years with a psychiatric hospital admission record for depression in Scotland between 2000 and 2019

	All (*N* = 28 808)	Male (*N* = 12 870)	Female (*N* = 15 938)
Age at admission, median (IQR) years	45 (32–64)	44 (32–61)	45 (32–66)
Follow-up, median (IQR) years	8.7 (3.8–14.3)	8.1 (3.5–11.9)	9.2 (4.1–14.7)
Person-years at risk	264 172	113 389	150 782
Deaths (%)	7903 (27.4)	3601 (28.0)	4302 (27.0)
Age at death, median (IQR) years	76 (59–84)	72 (54–81)	78 (64–86)

IQR, interquartile range.

**Fig. 1:**
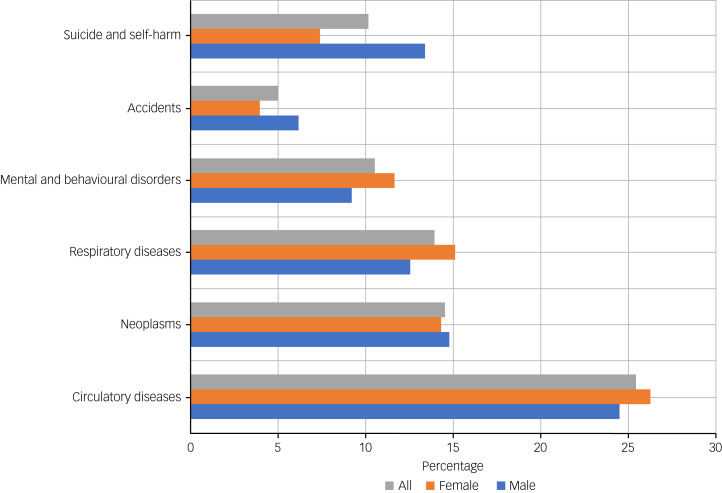
Cause-specific deaths among people with a psychiatric hospital admission record for depression in Scotland between 2000 and 2019.

Among those with severe depression, 6703 (84.8%) deaths were due to natural causes, with circulatory diseases (25.5%), neoplasms (14.5%) and respiratory diseases (14.0%) being the three most common causes of death for males and females combined. Mental and behavioural disorders accounted for 10.6% of deaths, with the majority of death causes in this category related to dementia. The remaining natural causes of death comprised smaller numbers of deaths across multiple disease categories and so were combined in a single ‘other’ category (Supplementary Table 2). Unnatural causes of death accounted for 15.2% of all deaths. Suicide, self-harm and injuries of undetermined intent accounted for the majority of these deaths (Fig. 1). Notably, the proportion of deaths from unnatural causes was higher in males than in females. Whereas the three most common causes of death in females were circulatory disease, neoplasms and respiratory disease, among males, self-harm and injuries of undetermined intent constituted the third leading cause of death after circulatory disease and neoplasms, accounting for 13.4% of deaths.

SMRs for all-cause and cause-specific mortality are presented for the entire cohort and by sex in Table 2. In comparison with all-cause mortality in the general Scottish population over the 20 year study period, the SMR for all-cause mortality among people with severe depression was 3.26 (95% CI 3.19–3.34). In sensitivity analyses, when we excluded 2163 people with psychiatric hospital admission records of comorbid schizophrenia, bipolar disorder, and drug- or alcohol-induced psychotic disorder from the depression cohort, the SMR decreased to 3.09 (95% CI 3.02–3.16; Supplementary Table 3). In the total cohort, circulatory diseases, the most common primary cause of death, had an SMR of 2.51 (95% CI 2.40–2.62), followed by neoplasms and respiratory diseases with SMRs of 1.69 (95% CI 1.59–1.79) and 3.79 (95% CI 3.56–4.01), respectively. Among natural causes of death, the excess rate of death was highest for mental and behavioural disorders (SMR 6.37, 95% CI 5.94–6.81). The SMR for unnatural causes was 10.0 (95% CI 9.44–10.6). The most common unnatural cause of death comprised suicide, self-harm and injuries of undetermined intent, which also had the largest SMR of all studied causes of death (15.2, 95% CI 14.1–16.2).

**Table 2:** All-cause and cause-specific numbers of deaths for people with a psychiatric hospital admission record for depression in Scotland between 2000 and 2019, and standardised mortality ratios (SMRs) by sex for comparison with the general population

	All			Male			Female		
	Observed deaths, *n*	Expected deaths, *n*	SMR (95% CI)	Observed deaths, *n*	Expected deaths, *n*	SMR (95% CI)	Observed deaths, *n*	Expected deaths, *n*	SMR (95% CI)
All causes	7903	2421	3.26 (3.19–3.34)	3601	1049	3.43 (3.32–3.54)	4302	1372	3.14 (3.04–3.23)
All natural causes	6703	2299	2.92 (2.85–2.99)	2892	972	2.98 (2.87–3.08)	3811	1327	2.87 (2.78–2.96)
Circulatory diseases	2020	806	2.51 (2.40–2.62)	886	343	2.58 (2.41–2.75)	1134	463	2.45 (2.31–2.59)
Neoplasms	1153	682	1.69 (1.59–1.79)	534	306	1.75 (1.60–1.89)	619	376	1.65 (1.52–1.78)
Respiratory diseases	1106	292	3.79 (3.56–4.01)	453	112	4.04 (3.67–4.42)	653	180	3.63 (3.35–3.91)
Mental and behavioural disorders^a^	835	131	6.37 (5.94–6.81)	333	52	6.40 (5.72–7.09)	502	79	6.35 (5.80–6.91)
Other natural diseases	1589	388	4.10 (3.89–4.30)	686	159	4.31 (3.99–4.64)	903	229	3.94 (3.69–4.20)
All natural causes	1200	122	10.00 (9.44–10.56)	709	77	9.21 (8.53–9.89)	491	45	10.91 (9.95–11.88)
Accidents (unintentional injuries)	395	69	5.72 (5.16–6.29)	224	38	5.89 (5.12–6.67)	171	31	5.52 (4.69–6.34)
Suicide, self-harm and injuries of undetermined intent	805	53	15.19 (14.14–16.24)	485	39	12.44 (11.33–13.54)	320	14	22.86 (20.35–25.36)

a. ICD-10 codes F00–F99; 70% dementia, 20% psychotic disorder due to psychoactive substance misuse, 10% other.

### Results for sex-stratified analyses

We found higher all-cause and cause-specific mortality in people with severe depression compared with the general population for both males and females (Table 2). For all-cause mortality, males had an SMR of 3.43 (95% CI 3.32–3.54) and females had an SMR of 3.14 (95% CI 3.04–3.23). SMRs for all natural causes combined and all individual natural causes of death were very similar in males and females. Males had an SMR of 9.21 (95% CI 8.53–9.89) and females 10.9 (95% CI 9.95–11.9) for unnatural causes of death. Mortality from suicide, self-harm and injuries of undetermined intent was more than 20 times higher in females with severe depression compared with females in the general Scottish population. This difference was less pronounced but still substantial among males (SMR 12.4, 95% CI 11.3–13.5).

### Circulatory disease subtype-specific mortality

IHD was the leading circulatory cause of death in the cohort with severe depression, followed by cerebrovascular disease. IHD-specific mortality was over two-fold greater in those with severe depression compared with the general population (SMR 2.23, 95% CI 2.09–2.38) (Table 3). Depression was associated with an even greater excess risk of cerebrovascular disease (SMR 3.00, 95% CI 2.77–3.22). When the results were stratified by sex, the SMR for cerebrovascular diseases was higher in males (3.42, 95% CI 3.00–3.84) than in females (2.79, 95% CI 2.53–3.06), although the confidence intervals overlapped slightly. The number of observed deaths was at least 2.5 times higher than expected for the categories of other cerebrovascular diseases and other CVDs. The SMRs for other CVDs were higher in females than in males, but numbers of deaths were small, and the confidence intervals overlapped.

**Table 3:** Circulatory disease subtype-specific mortality of people with a psychiatric hospital admission record for depression in Scotland between 2000 and 2019

	All			Male			Female		
	Observed deaths, *n*	Expected deaths, *n*	SMR (95% CI)	Observed deaths, *n*	Expected deaths, *n*	SMR (95% CI)	Observed deaths, *n*	Expected deaths, *n*	SMR (95% CI)
Ischaemic heart disease	906	406	2.23 (2.09–2.38)	477	203	2.35 (2.14–2.56)	429	203	2.11 (1.91–2.31)
Cerebrovascular disease	686	229	3.00 (2.77–3.22)	253	74	3.42 (3.00–3.84)	433	155	2.79 (2.53–3.06)
Other cerebrovascular diseases	291	117	2.49 (2.20–2.77)	101	43	2.35 (1.89–2.81)	190	74	2.57 (2.93–2.20)
Other cardiovascular diseases	137	54	2.54 (2.11–2.96)	55	24	2.29 (1.69–2.90)	82	30	2.73 (2.14–3.32)

SMR, standardised mortality ratio.

## Discussion

### Main findings

We found that people with a psychiatric hospital admission record for depression in Scotland had a more than three-fold increased risk of all-cause mortality. Severe depression was associated with three-fold and ten-fold increased risks of deaths from natural and unnatural causes of death, respectively, compared with the general population. Although the relative excess death was greatest for unnatural causes of death, deaths from circulatory disease, neoplasms and respiratory disease were the most common causes of death. Among these leading causes of death, the excess relative mortality was highest for circulatory and respiratory diseases. Among circulatory deaths, the excess relative mortality was largest for cerebrovascular-specific deaths. Although patterns were similar in males and females for all-cause mortality and, in general, for cause-specific mortality, the excess relative mortality from suicide and self-harm was markedly higher in females than in males.

### Comparison with findings from other studies

Previous studies have provided limited insight into cause-specific mortality among people with depression specifically. The majority of previous studies have combined several types of mental illness,^5,7,10^ making it challenging to understand the specific association between depression and mortality. Our findings provide understanding of the association between severe depression and cause-specific mortality by using a more comprehensive list of causes of death, including subtypes of circulatory disease. A previous study conducted in Scotland between 1986 and 2009 reported an SMR of 2.01 (95% CI 1.96–2.05) for all-cause mortality among individuals with depression identified from psychiatric hospital admission records. This was lower than our comparable estimate for 2000 to 2019 of 3.09 (95% CI 3.02–3.16) from the sensitivity analysis after excluding people with other SMI, suggesting that the relative effects of severe depression on mortality may have increased over time.^9^ The expected number of deaths in the previous Scottish study was roughly 20% higher than our estimate, which is unsurprising given the improvement in general population mortality rates over time.^17^

Two other studies have previously reported on all-cause mortality and a few specific causes of death among people with depression identified from primary and secondary care records.^6,8^ Two studies conducted in Italy^6^ and Taiwan,^8^ with 10 year and 3 year follow-up periods, respectively, reported lower SMRs for all-cause mortality in people with depression than that found in our study (SMR 1.89 [95% CI 1.84–1.94] and 1.83 [95% CI 1.81–1.86], respectively). Direct comparison of relative mortality to these studies was not appropriate owing to differences in reference populations. Nonetheless, these studies consistently reported that composite SMI^7^ or depression^6,8^ was associated with a higher risk of mortality relative to the general population, with CVD and suicide being the most common natural and unnatural causes, respectively. In the UK, a Welsh study that included 29 797 individuals with SMI, including schizophrenia, bipolar disorder, or other mood-related disorders and other non-organic psychotic disorders identified from primary and/or secondary care records, reported broadly similar findings to those of our study.^7^ However, direct comparisons are difficult as the Welsh study included depression within a composite mood disorder category. During the study period of 2004 and 2013, 1565 deaths occurred in this group, with a lower all-cause SMR (2.2, 95% CI 2.1–2.3) than that observed in our study for depression only. This is likely to be due to the ascertainment of mood disorder from both primary and secondary care records. The authors reported a higher all-cause SMR for all SMI conditions combined when restricting analyses to participants with hospital admission records as the only source of SMI diagnosis; this aligned with the SMR for depression and all-cause mortality in our study. As in our study, SMRs for all-cause mortality were slightly higher in males than females. Comparisons of cause-specific mortality are also difficult owing to the Welsh study reporting on cause-specific mortality for all SMI conditions combined.^7^ However, the overall patterns of results were broadly similar, including findings by sex. SMRs for natural-cause-specific deaths were generally higher in males than in females (albeit with confidence intervals often overlapping). There were, however, differences in the magnitude of the sex-specific SMRs across various specific causes of death, including respiratory disease, cerebrovascular disease and, for women only, mental and behavioural disorders. The SMRs for these causes of death in our study were notably higher (with confidence intervals often not overlapping) than those in the Welsh study. For example, although SMRs for IHD were similar across the two studies, the overall SMR for cerebrovascular disease was greater in our study (SMR 3.0, 95% CI 2.77–3.22) than in the Welsh study (SMR 2.3, 95% CI 2.1–2.6). Finally, the higher relative risk of death from suicide, self-harm and undetermined intent among females compared with males aligned with findings from the Welsh study,^7^ and from the Taiwanese study, which reported similar findings for depression in relation to suicide.^8^ In Scotland, this sex difference is largely explained by the higher absolute suicide mortality rates in males compared with females in the general Scottish population (22.8 deaths from suicide per 100 000 males *v.* 7.5 for females in 2019).^18^

### Strengths and limitations

Our study had several strengths. To our knowledge, this is the first study to describe all-cause and cause-specific mortality by sex in people with severe depression using a reasonably comprehensive list of causes of death. We were able to use large-scale national linked data over a recent 20 year period, allowing for a longer follow-up time from admission to mortality than the majority of previous studies. Moreover, we included people with a diagnosis of depression from psychiatric hospital admission records regardless of diagnosis with other mental disorders, with a sensitivity analysis among people with depression in the absence of other SMI diagnoses. Our study expands on a previous Scottish study^9^ to provide additional insight into the mortality experience of people with depression specifically, by examining cause-specific death in far more detail and using contemporaneous data.

A key limitation of our study was that it was based on people who were admitted to hospital and so were likely to have more severe depression than people whose depression was diagnosed in primary care or out-patient clinics. Although this does not limit the internal validity of our study, our findings are not likely to be generalisable to the wider population of people with a diagnosis of depression. A small positive dose–response relationship has previously been described between severity of depression and premature mortality, with increased risk of mortality still observed for less severe depression.^19^ Second, although the SMR is a useful measure of mortality on a population level, it is a relative measure and therefore cannot be used to make direct comparisons with other settings or populations. Finally, it was outside the scope of our study to examine mortality trends over time or to investigate the potential effect of multiple hospital admissions as a further proxy for severity of depression.

### Interpretation and implications

The mechanisms underlying the association between depression and mortality are not fully understood but are complex and multifactorial. Depression has been linked to increased risk of various chronic physical conditions, including CVD, respiratory diseases and diabetes, all of which can increase the risk of mortality.^20–22^ In our study, among people with severe depression, the most common cause of death was circulatory disease, followed by neoplasms and respiratory diseases, similar to the pattern observed in the general Scottish population.^17^ Depression is associated with a range of behavioural and lifestyle factors that can affect physical health, such as unhealthy diet, lack of exercise and substance misuse.^20^ In addition, people with depression may be less likely to receive optimal medical care or adhere to treatment for physical health conditions.^3^ In Scotland, as in other settings, severe depression is associated with a 22% reduction in receipt of coronary revascularisation following a myocardial infarction.^23^

Despite the introduction of suicide prevention strategies in Scotland over the past 20 years,^24^ our findings suggest that the risk of death from suicide among people with severe depression remains markedly higher than that of the general population. Several strategies have been proposed to address the increased mortality risk associated with depression and other SMI.^20,25–27^ Many of these strategies aim to prevent the onset of mental disorders, reduce case fatality and optimise prognosis, particularly among those with coexisting physical diseases. In general, interventions in the proposed strategies included improving access to evidence-based treatments, adopting collaborative care models, addressing social determinants of health and advocating for policy changes to reduce disparities in health outcomes between people with and without mental health disorders.^20,25–27^

In some countries, these strategies have been helpful in improving general health outcomes in people with mental health disorders, including improved cardiovascular risk profiles and lower suicide rates.^20,26,27^ Despite widespread recognition of the excess risks of physical diseases and premature mortality observed in individuals with mental illness over recent decades, it is disappointing to report evidence of a possible widening gap in mortality between people with severe depression compared with the general population of Scotland. Further investigation is needed to establish whether the mortality gap has indeed widened in this setting and for which specific causes of death.

### Summary and future directions

In Scotland, compared with the general population, people with severe depression have a substantially higher risk of all-cause mortality, with relative mortality varying by specific cause of death. Relative mortality is similar by sex, except for deaths by suicide, self-harm and injuries of undetermined intent. Further research is needed to identify the contributions of socioeconomic status, age, physical disease and other factors, including time trends, to the higher risk of all-cause and cause-specific mortality among people with severe depression. In addition, further insight from qualitative research would help to identify barriers and enablers to delivering optimal physical healthcare to this vulnerable group. Future healthcare models should ensure equitable and optimal provision of care for the physical health of people with severe depression and include strategies to reduce the burden of preventable death in this high-risk population group.

## Supporting information

Alotaibi et al. supplementary material Alotaibi et al. supplementary material

## Data Availability

Data-sets can be requested by contacting the electronic Public Health Scotland Data Research and Innovation Service (eDRIS). Details of the available data-sets and the application process are available from https://www.isdscotland.org/Products-and-Services/eDRIS/.
