# Supplementary material for: Severe depression and all-cause and cause-specific mortality in Scotland: 20 year national cohort study
Source: BJPsych Open. 2024 Jan 11;10(1):e28. doi: 10.1192/bjo.2023.633 (PMC10790224; doi:10.1192/bjo.2023.633)
Supplement: Alotaibi et al. supplementary material [file S2056472423006336sup001.docx]

**Table S1: ICD-10 codes used to identify mental health conditions and causes of death.**

| **Mental health condition** | **ICD10 code**  **(First 3 digits)** |
| --- | --- |
| Schizophrenia | F20- F29 |
| Bipolar disorder | F30- F31 |
| Drug or alcohol-induced psychotic disorder | F10-F19 |
| Depression | F32-F33 |
| **Underlying cause of death** |  |
| Diseases of the circulatory system | I00-I99 |
| Neoplasms | C00-D48 |
| Diseases of the respiratory system | J00-J99 |
| Mental and behavioural disorders | F00-F99 |
| Accidents (unintentional injuries) | V01-X59, Y85-Y86 |
| Suicide, self-harm and injuries of undetermined intent | X60-X84, Y10-Y34, Y870, Y872 |
| **Circulatory diseases subcategories** |  |
| Ischemic heart disease | I20-I25 |
| Cerebrovascular disease | I60-I69 |
| Other cerebrovascular diseases | I100-I13, I26-I51 |
| Other cardiovascular diseases | I70-I99 |

**ICD: International classification of diseases**

**Table S2: Distribution of other natural causes of death in people with a psychiatric hospital admission record for depression in Scotland 2000-2019**

| Cause (ICD-10 code) | **N (%)** |
| --- | --- |
| Certain infectious and parasitic diseases (A00-B99) | 102 (6.42) |
| Diseases of the blood and blood-forming organs and certain disorders involving the immune mechanism (D50-D89) | 22 (1.38) |
| Endocrine, nutritional and metabolic diseases (E00-E90) | 162 (10.20) |
| Diseases of the nervous system (G00-G99) | 439 (27.63) |
| Diseases of the digestive system (K00-K93) | 467 (29.39) |
| Diseases of the skin and subcutaneous tissue (L00-L99) | 18 (1.13) |
| Diseases of the musculoskeletal system and connective tissue (M00-M99) | 38 (2.39 |
| Diseases of the genitourinary system (N00-N99) | 163 (10.26) |
| Pregnancy, childbirth and the puerperium (O00-O99) | 1 (0.06) |
| Congenital malformations, deformations and chromosomal abnormalities (Q00-Q99) | 11 (0.69) |
| Symptoms, signs and abnormal clinical and laboratory findings, not elsewhere classified (R00-R99) | 166 (10.45) |
| **Total** | **1589** |

**Table S3: Sensitivity analysis for all-cause mortality of people with a psychiatric hospital admission record for depression after excluding people who also had admission records for schizophrenia, bipolar disorder or drug or alcohol-induced psychotic disorder in Scotland 2000-2019**

|  | **Population** | **Person-years** | **Observed deaths, n** | **Expected deaths, n** | **SMR (95% CI)** |
| --- | --- | --- | --- | --- | --- |
| **All** | 26645 | 242,130 | 7,364 | 2,385 | 3.09 (3.02- 3.16) |
| **Female** | 15,009 | 141,177 | 4,106 | 1,377 | 2.98 (2.99- 3.18) |
| **Male** | 11,550 | 100,953 | 3,258 | 1,008 | 3.23 (3.12- 3.34) |

**CI: Confidence interval; SMR: Standardised mortality ratio**
